# Promoting health and productivity management in small companies through outreach-based public-private partnership: the Yokohama Linkworker Project

**DOI:** 10.3389/fpubh.2024.1345771

**Published:** 2024-07-05

**Authors:** Yuko Kai, Yuya Fujii, Naoki Takashi, Kaori Yoshiba, Yuko Muramatsu-Noguchi, Takayuki Noda, Takashi Jindo, Tetsuhiro Kidokoro, Yoko Yajima, Junko Kasuga, Takashi Arao

**Affiliations:** ^1^Physical Fitness Research Institute, Meiji Yasuda Life Foundation of Health and Welfare, Tokyo, Japan; ^2^Department of Health Economics, Center for Gerontology and Social Science, National Center for Geriatrics and Gerontology, Aichi, Japan; ^3^Division of Art, Music, and Physical Education, Osaka Kyoiku University, Osaka, Japan; ^4^Faculty of Sport Science, Nippon Sport Science University, Tokyo, Japan; ^5^Health Promotion Division, Health and Social Welfare Bureau, Yokohama City Hall, Kanagawa, Japan; ^6^Health Insurance Bureau, Ministry of Health, Labour and Welfare, Tokyo, Japan

**Keywords:** occupational health, health disparity, small companies, workplace health promotion, health and productivity management, public-private partnership

## Abstract

**Introduction:**

With health promotion initiatives in small companies lagging behind those in larger corporations, strengthening health and productivity management in small companies through innovative strategies is an urgent priority. We hypothesized that an outreach strategy involving a public-private partnership would be beneficial for this purpose. The present study examines the implementation of a public-private partnership strategy in Yokohama City, Japan, assessing its impact on health and productivity management in small enterprises, focusing on implementation outcomes.

**Methods:**

As part of the Yokohama Linkworker Project (Y-Link Project), this study describes and examines a public-private partnership program in Yokohama City, Japan, involving the city’s government and a private life insurance company. Trained insurance sales representatives served as “Linkworkers” for the program, reaching out to small enterprises in the city. These Linkworkers provided tailored support to these companies, assisting them with obtaining the “Yokohama Health and Productivity Management Certification” issued by the City of Yokohama authorities and collaborating with external entities to offer health promotion programs for employees. Program interventions took place from August to September 2020. The RE-AIM framework was utilized to evaluate the Project. Data were extracted from Linkworkers’ activity records, certification records, the Linkworker survey, and follow-up surveys with participating companies at 6– and 18– months post-interventions.

**Results:**

Within 2 months, 71 Linkworkers visited 500 companies (50% were small firms, <50 employees). Among them, 224 (45%) enterprises received certifications, contributing to an increased regional certification rate. Linkworker-assisted companies tended to be significantly smaller in size. The odds ratios of implementing workplace health promotion programs in certified firms, compared to non-certified firms, were 4.09 (95% CI: 1.79–9.35) at 6 months and 2.31 (95% CI: 1.04–5.11) at 18 months. For small firms, the odds ratios were 6.87 (95% CI: 1.74–27.06) at 6 months and 3.42 (95% CI: 1.17–10.03) at 18 months. The certification retention rate at 24 months was 60%, irrespective of company size. Linkworkers perceived the outreach strategy as having a positive impact on their primary operations.

**Conclusion:**

The Y-Link Project’s outreach strategy enhanced health and productivity management in small enterprises in Yokohama City, enabling long-term health promotion programs addressing program availability disparities related to company size.

## 1 Introduction

The workplace is an important setting for health promotion; however, efforts are inequitable based on company size. Workplace health promotion programs that promote employee health and productivity (1) are not implemented as often in small companies as in larger ones (2–5). Employees in small companies experience socioeconomic disadvantages, leading to poorer health (6) and a higher mortality risk in old age (7). Health promotion should be adopted in small-scale companies to correct company size-related health disparities in employee health and productivity.

Employee health promotion also benefits employers by reducing healthcare costs and boosting stock prices (8, 9). The employer’s investment in employee health promotion is called health and productivity management (HPM) (10). HPM is important in a society with a declining birthrate and an aging population (11). The national award systems in the United States (12), Wales (13), and elsewhere support HPM promotion. Japan has followed suit, recognizing that securing a productive labor force while reducing medical costs are important goals for a nation that is experiencing a rapid decline in birthrate and a population that is aging (14, 15).

Overall, companies implementing HPM have reported better employee health status (16) and higher stock prices (17). However, the uptake of HPM has varied by company size. For example, even though the number of large companies implementing HPM has increased, similar program interventions in smaller companies have not, due primarily to these small businesses’ limited capacity to invest in these types of workplace interventions (11). HPM in small companies could enable a reduction in health disparities caused by company size. Yet, to the best of our knowledge, no specific methods or program strategy have been established to promote HPM in these small companies.

Public institutions seeking to promote HPM can create certification programs (15) and award outreach efforts (13); they can also conduct media campaigns about HPM interventions. However, only large companies typically respond to such campaigns, as these public sector actions alone are insufficient to promote and facilitate implementation of HPM in small companies. With cooperation from private sector, public institutions like a city government may be able to do much more, especially when it comes to delivering messaging and information for small companies to consume and trust (18).

In recent years, cooperation between public institutions and private companies in the health sector has become common and is regarded as a public-private partnership (PPP) strategy that could effectively engage private/commercial sector entities (19, 20). Many PPPs in primary healthcare, for example, have increased access to prevention and health services (21). Insurance brokers with small business clients often have greater capacity to work with them and represents a potential bridge between small business owners/companies and outside wellness programs (18). As such, an outreach strategy based on a PPP involving a local government and a private insurance company could be a novel and impactful approach to promoting HPM in small companies—this is the primary hypothesis of the present program evaluation.

The present program evaluation sought to assess the implementation and impact of a novel PPP strategy used to promote HPM in Yokohama, City Japan. This particular strategy and its program interventions targeted micro-and small companies with 49 or fewer employees. Under Japanese law, such small companies are legally exempted from appointing industrial physicians and holding health committee meetings. We evaluated this innovative strategy using the RE-AIM framework— domains include reach, effectiveness, adoption, implementation, and maintenance— (22) to systematically assess the feasibility and implementation progress of these HPM interventions in the small company setting in the real-world.

## 2 Materials and methods

### 2.1 Study design and setting

**Figure 1:**
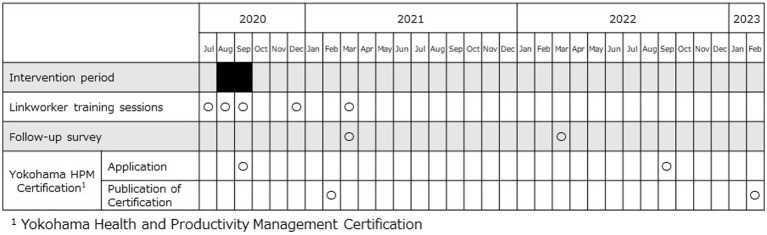
Schedule of the study.

Conducted as part of the Yokohama Linkworker Project (Y-Link Project), this study describes and examines a PPP strategy in Yokohama City, Japan; the program involves the city’s government (the City of Yokohama) and a private life insurance company (Company M). Yokohama City, a large commercial city with a population of approximately 3.76 million, actively promotes the HPM certification program. Company M, a mutual life insurance company with around 47,000 employees and branch offices nationwide, including 41 sales offices in Yokohama City with around 2,000 employees, participated in the project. The primary aim of the Y-Link Project is to promote HPM in Yokohama City companies and encourage health promotion among the city’s citizens. The project commenced in August 2020 and is still ongoing, following a partnership agreement signed by the City of Yokohama and Company M in March 2020. During the intervention period (from August to September 2020; Figure 1), the sales staff from Company M were selected as “Linkworkers” and trained to provide interventions on a company-by-company basis. These trained Linkworkers visited client companies to recommend HPM. Data collection on intervention companies continued until February 2023. Although the Linkworker activities were systematically implemented during the intervention period, the necessity of engaging in post-intervention follow-up activities was decided by each Linkworker.

### 2.2 Intervention strategy

Linkworkers recommended client companies to pursue the “Yokohama Health and Productivity Management Certification (Y-HPM Certification),” a system certified by the City of Yokohama, as an intervention method for promoting HPM. The certification system, launched in 2017, recognizes companies in Yokohama City that engage in health management. Companies seeking certification must apply to the City of Yokohama in September of the previous year, and the results of the examination are announced in February. The examination criteria include a declaration of systematic HPM implementation, establishment of a promotion system, understanding employee health issues, and implementation and evaluation of WHPPs. The Y-HPM Certification has three levels, with the easiest level available to companies that declare their commitment to the implementation of HPM. Once certified, a company becomes eligible to use the certification mark, publicize it on the city’s website, and receive support and subsidies for its HPM efforts. Financial benefits include favorable loan terms and guarantee fee subsidies. Additionally, certified companies receive preferential treatment in public procurement by the City of Yokohama. Essentially, certified companies receive social recognition, support for health promotion, and economic benefits through Y-HPM Certification. Certification is valid for 2 years; therefore, companies must reapply and undergo an examination every 2 years to maintain their certification.

The companies visited were primarily selected by the Linkworkers from among their existing client companies. Some visits involved follow-up activities, such as reviewing insurance policies, whereas others were simply seasonal greetings. Additionally, there were visits to companies where appointments were made spontaneously with company representatives. During client visits, Linkworkers began by providing explanations about health management and the Y-HPM Certification. They then offered to assist interested companies with their certification applications. Additionally, Linkworkers introduced external public institutions that support WHPPs for companies wishing to implement specific WHPPs for their employees, and helped with applications as needed. These interventions were tailored to meet the specific needs of each client. Linkworker activities were conducted within Company M’s working hours, and no additional salary was provided. To ensure the proper use of Linkworker activities and prevent their use in sales activities, the City of Yokohama and Company M established the “Linkworker Activity Guideline.”

### 2.3 Linkworker training program

The City of Yokohama and Company M collaborated to train Linkworkers. The group training session was held three times: once before the intervention and twice during the intervention. Additionally, two online follow-up sessions were conducted after the intervention period. A total of 71 corporate sales representatives and sales office managers from 41 Company M offices in Yokohama City participated in the program. The training session included a discussion on the importance of HPM, information on the Y-HPM Certification application process, different external organizations supporting WHPPs, and an opinion exchange workshop. During the follow-up sessions, successful cases were reported, and the results of the Y-HPM Certification were announced. The first session lasted for 180 min, while the subsequent sessions were 90 min each.

### 2.4 Evaluation methods

In this study, we conducted an assessment using the RE-AIM framework, which has been widely applied in public health and implementation science for 20 years. The framework bridges the research-to-practice gap and addresses health inequities. Recognized for its transparent reporting, it serves as a valuable tool for strategic planning in disseminating evidence-based interventions (22). The RE-AIM framework comprises five dimensions: reach, effectiveness, adoption, implementation, and maintenance. In this intervention, it was elucidated through the following methods.

“Reach” was examined by analyzing the number and characteristics of client companies visited by Linkworkers during the intervention period. “Effectiveness” was evaluated at two levels: intervention and community. At the intervention level, we assessed the proportion of companies successfully obtaining Y-HPM Certification among those where Linkworkers intervened. At the community level, changes in the number of companies receiving Y-HPM Certification throughout Yokohama City served as indicators of intervention impact. “Adoption” was measured by evaluating the project’s participation rate among Company M’s sales offices in Yokohama City. Additionally, feedback regarding Linkworkers from intervention companies was collected from Yokohama City officials. “Implementation” was assessed by examining the proportion of companies visited by Linkworkers that implemented the intervention. Furthermore, we investigated the specific content of the interventions provided. “Maintenance” was determined by evaluating the implementation status of the WHPPs for employees at 6- and 18-months post-intervention. To gauge the maintenance of Y-HPM Certification, we examined the companies’ re-certification statuses 24 months after their initial certification. Additionally, we investigated the perceptions of Linkworkers regarding the impact on primary operations, to verify whether this innovative strategy can be sustained by private enterprises.

### 2.5 Key constructs and definitions

Company sizes were categorized by number of employees into “micro” (<5 employees), “small” (5–49 employees), “medium” (50–299 employees), and “large” (≥300 employees) (23). In Japan, companies with 50 or more employees are legally obligated to appoint an industrial physician and hold health committee meetings, but companies with 49 or fewer employees are exempted from this rule; hence, the health management in the latter companies tends to be inadequate. Therefore, we focused on evaluating micro-and small companies.

### 2.6 Data sources and collection

We extracted details from the activity records of Linkworkers, which detailed information about the companies they had visited. This included data on company size, industry type, certification status, and the specifics of the interventions. Data on the number of companies that obtained Y-HPM Certification at the community level and their scale was obtained from the City of Yokohama.

Additionally, we conducted follow-up mail surveys of the companies where interventions had occurred at two time points after the initial intervention—6 months (March 2021) and 18 months (March 2022). The surveys delved into the implementation status of WHPPs related to lifestyle improvement (“implementing,” “considering implementation,” or “not implemented”), the level of awareness of the HPM at the time of the Linkworker intervention, and impact of the coronavirus disease 2019 (COVID-19) pandemic on business performance. Each of these variables was identified using the following questions: *“Does your company plan to implement initiatives to improve the physical activity and dietary habits of your employees (*e.g.*, walking and eating campaigns, lifestyle improvement seminars, introduction of apps,* etc.*)?, “Did you know the term ‘health and productivity management’?,”* and *“Has the COVID-19 epidemic affected your company’s performance?”* This follow-up survey aimed to include all visited companies, with responses ideally provided by company presidents or individuals responsible for health management. However, survey questionnaires were not sent to those companies that had previously declined the survey through Linkworkers. The follow-up survey response rates were 56% at 6 months and 51% at 18 months post-intervention. The differences in the characteristics of responded and non-responded companies are detailed in Supplementary Tables S1, S2.

In all sessions of the Linkworker training program except for the first session, an anonymous survey was conducted among the Linkworkers to assess the impact of their activities on primary operations. The question posed was, “What impact do Linkworker activities have on primary operations?” with the response options including “positive impact,” “somewhat positive impact,” “neutral impact,” “somewhat negative impact,” and “negative impact.” Moreover, in the surveys conducted during the third and fourth sessions, the Linkworkers were asked to provide reasons for their selected choice in free-text format. The response rate for the Linkworker survey ranged from 48 to 87%, with an average of 70%.

### 2.7 Data analysis

The χ^2^ test and residual analysis were used to assess the differences in characteristics between certified and non-certified companies, size differences among companies with Y-HPM Certification and the responses from the Linkworker survey. The “implementing” and “considering implementation” responses to the follow-up survey evaluating the impact of Y-HPM Certification on WHPP adoption were re-classified as “WHPPs implemented.” A multiple logistic regression analysis was conducted, with “WHPPs implemented” as the dependent variable and company size, industry type, employment status, and impact of COVID-19 as moderator variables. Sub-analyses were only conducted in micro-and small companies as the sample size from medium and large companies was insufficient. The statistical significance level was set to <5%, and SPSS Statistics version 26 (IBM) was used to perform all analyses. Content analysis was employed to extract categories from the free-text responses in the Linkworker survey.

### 2.8 Ethics statement

This study was approved by the ethics committee of the Physical Fitness Research Institute, Meiji Yasuda Life Foundation of Health and Welfare (approval number: 2020–0002). The research collaboration between the City of Yokohama and the Physical Fitness Research Institute is governed by a memorandum of understanding, ensuring that the Institute offers academic support and scientific evaluation for the Y-Link Project.

## 3 Results

### 3.1 Reach

**Figure 2:**
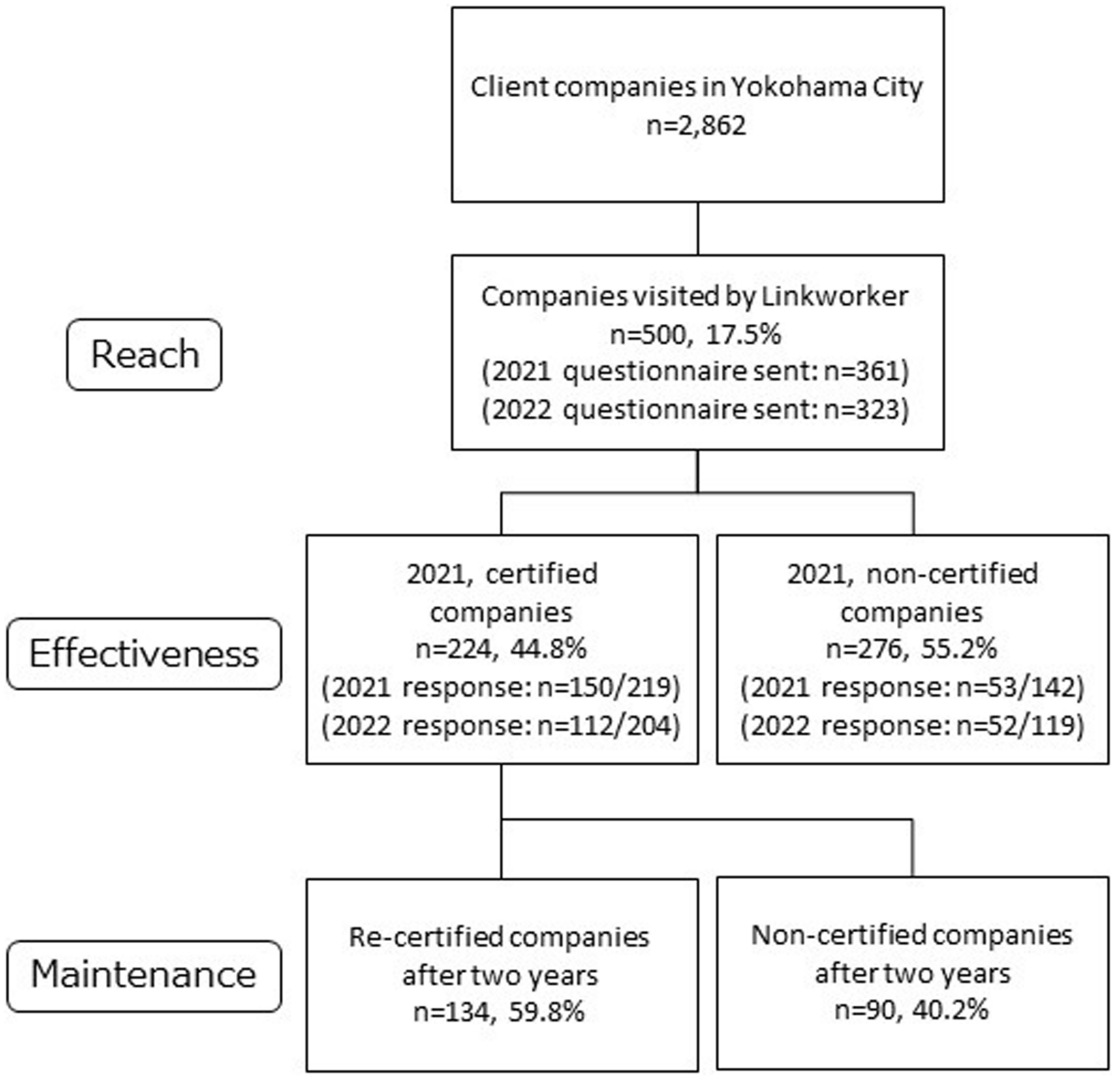
Flow chart of the process of selecting the intervening companies.

Of the 2,862 client companies of Company M in Yokohama City, Linkworkers visited 500 during the intervention period, achieving a 2-month reach rate of 18% (Figure 2). Of these, 50% were micro-and small companies. When companies of an unknown size are excluded from the 500 companies visited the proportion of micro-and small companies increases to 69% of the total (Table 1). The type of industry each company operated in was not controlled for in this study; nevertheless, the visited companies included a diverse range of industries without notable bias. The majority of the companies had full-time regular employees. Many companies were unaware of the HPM at the time of Linkworker intervention.

**Table 1:** Characteristics of the intervening companies.

	all		Certified companies		Non-certified companies		*p* value^a^
	*n* = 500		*n* = 224		*n* = 276		
Company size							0.02
Micro (<5 employees)	62	(12.4)	40	(17.9)	22	(8.0)	
Small (5–49 employees)	187	(37.4)	119	(53.1)	68	(24.6)	
Medium (50–299 employees)	95	(19.0)	46	(20.5)	49	(17.8)	
Large (≥300 employees)	16	(3.2)	6	(2.7)	10	(3.6)	
Unknown	140	(28.0)	13	(5.8)	127	(46.0)	
Industry type							0.41
Manufacturing	58	(11.6)	29	(12.9)	29	(10.5)	
Service	55	(11.0)	38	(17.0)	17	(6.2)	
Construction	54	(10.8)	33	(14.7)	21	(7.6)	
Transportation/postal business	38	(7.6)	22	(9.8)	16	(5.8)	
Other	149	(29.8)	88	(39.3)	61	(22.1)	
Unknown	146	(29.2)	14	(6.3)	132	(47.8)	
Employment type							0.35
Mainly full-time employees	306	(61.2)	173	(77.2)	133	(48.2)	
Half are non-regular	46	(9.2)	29	(12.9)	17	(6.2)	
Mainly non-regular employees	9	(1.8)	7	(3.1)	2	(0.7)	
Unknown	139	(27.8)	15	(6.7)	124	(44.9)	
Awareness of HPM^b^							0.62
Content known	51	(10.2)	38	(17.0)	13	(4.7)	
Had heard of it but did not know content	55	(11.0)	38	(17.0)	17	(6.2)	
Did not know	97	(19.4)	74	(33.0)	23	(8.3)	
Unknown	297	(59.4)	74	(33.0)	223	(80.8)	

a Comparison of certified and non-certified companies using the chi-square test (excluding unknown companies).

b HPM, Health and productivity management.

### 3.2 Effectiveness: intervention level

Of the 500 companies reached by Linkworkers, 224 obtained Y-HPM Certification, resulting in a success rate of 45% for the Linkworker interventions. Certified companies were significantly smaller than non-certified companies (Table 1). No significant differences were observed in the industry type, employment type, and awareness of HPM.

### 3.3 Effectiveness: community level

**Figure 3:**
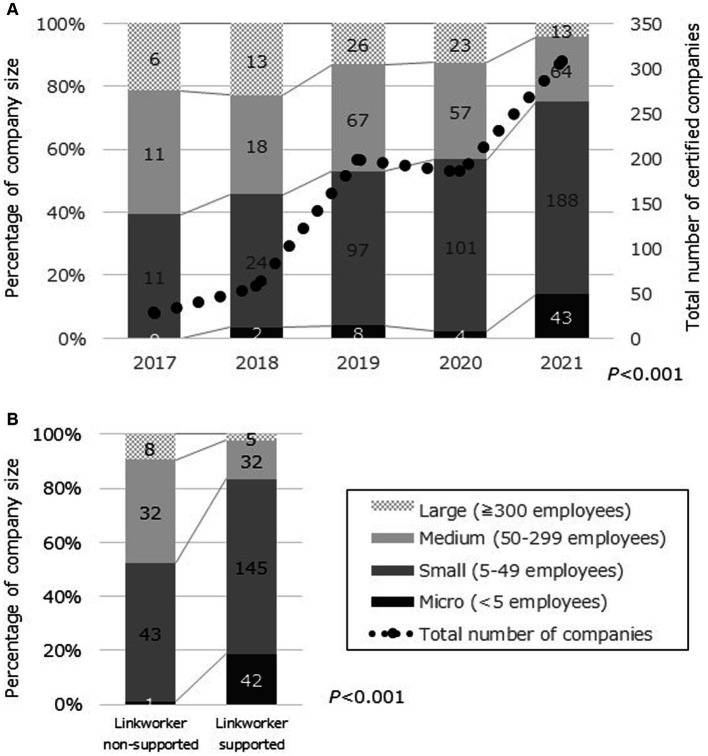
Changes in the number of certified companies at community level. **(A)** Total number and size of certified companies in Y-HPM Certification^1^ from 2017 to 2021. **(B)** Comparison of company size with and without Linkworker support among Y-HPM Certification^1^ companies in 2021. ^1^Y-HPM, Yokohama Health and Productivity Management Certification.

Figure 3 illustrates the evolution of companies awarded the Y-HPM Certification over a 5-year period, starting from its initiation in 2017. The impact of this project’s intervention is solely observed in 2021, as Linkworker support to companies was implemented in the preceding year, 2020. In 2021, the number of certified companies reached a record high of 308, compared to previous years (Figure 3A). A significant difference was observed in the trend of company size proportions. Residual analysis indicated that there were significantly fewer large and medium-sized companies and significantly more small and micro-sized companies in 2021 compared with other years. The proportion of micro-and small-sized companies increased by 18.2 percentage points (from 57 to 75%). Among the 224 companies supported by Linkworkers, there was a significant size difference compared with the 84 unsupported companies (Figure 3B). Residual analysis for Linkworker-supported enterprises revealed a significantly lower proportion of large and medium businesses and a significantly higher proportion of small and micro businesses compared with the unsupported group. The percentage of micro-and small-sized companies was 30.1 points higher than the unsupported group (52% vs. 84%).

### 3.4 Adoption

Company M’s 41 sales offices in Yokohama City completely adopted the Linkworker program (adoption rate: 100%). The intervention companies had no complaints regarding the Linkworkers.

### 3.5 Implementation

Linkworkers implemented the prescribed HPM support activities in 317 of the 500 visited companies (implementation rate: 63%). Activities included HPM explanation (100%), information/advice certification acquisition (51%), application form support (42%), and application submission (42%).

### 3.6 Maintenance

We assessed the Y-HPM Certification’s impact on the implementation of WHPPs for improvements of employees’ lifestyles. Certified companies showed a significantly higher odds ratio (2.31–4.09) of implementing WHPPs compared with non-certified ones (Table 2). The odds ratio (3.24–6.87) was higher in the sub-analysis of micro-and small companies (<49 employees). Out of the 224 certified companies in 2021, 134 were re-certified after 24 months (maintenance rate: 60%; Figure 2). No significant differences (*p* = 0.51) were observed in the maintenance rate by company size (<49 employees: 61%, ≥50 employees: 54%).

**Table 2:** Multiple logistic regression analysis: impact of the Y-Link Project on implementation of workplace health promotion programs.

	n	case	%	OR	95%CI	*p* value^a^
6 months						
All companies						
Non-certified companies	53	16	30.2	1.00		
Certified companies	148	89	60.1	**4.09**	(1.79 − 9.35)	<0.01
Companies with 49 or fewer employees						
Non-certified companies	23	5	21.7	1.00		
Certified companies	102	56	54.9	**6.87**	(1.74 − 27.06)	<0.01
18 months						
All companies						
Non-certified companies	51	23	45.1	1.00		
Certified companies	111	65	58.6	**2.31**	(1.04 − 5.11)	0.04
Companies with 49 or fewer employees						
Non-certified companies	30	11	36.7	1.00		
Certified companies	81	42	51.9	**3.42**	(1.17 − 10.03)	0.03

a Adjusted for company size, industry type, employment type, and impact of the coronavirus disease 2019 pandemic. CI, confidence interval; OR, odds ratio. Bold indicates statistical significance.

In Table 3, we present the impact of Linkworker activities on primary operations as self-evaluated by Linkworkers. The majority of Linkworkers reported (somewhat) positive impacts on primary operations, with no Linkworkers selecting negative impacts in any of the surveys. The main reasons cited for selecting (somewhat) positive impacts, as extracted through content analysis, included “contribution to clients,” “promotion of business activities,” “strengthening relationships with clients,” “improvement in the quality of customer service,” “enhancement of the company’s social reputation,” and “increase in customer satisfaction.” Reasons for selecting somewhat negative impacts included “burden on primary operations,” “insufficient customer demand,” and “lack of time due to the Covid-19 pandemic.”

**Table 3:** Impact of Linkworker activities self-evaluated by Linkworkers on primary operations.

	2nd session		3rd session		4th session		5th session		*p* ^a^
	*n* = 51		*n* = 62		*n* = 52		*n* = 34		
Positive impact	12	(23.5)	13	(21.0)	14	(26.9)	7	(20.6)	0.34
Somewhat positive impact	14	(27.5)	30	(48.4)	26	(50.0)	21	(61.8)	
Neutral impact	9	(17.6)	7	(11.3)	8	(15.4)	4	(11.8)	
Somewhat negative impact	6	(11.8)	5	(8.1)	2	(3.8)	1	(2.9)	
Negative impact	0	(0.0)	0	(0.0)	0	(0.0)	0	(0.0)	
Unknown	10	(19.6)	7	(11.3)	2	(3.8)	1	(2.9)	

a Using the chi-square test (excluding unknown).

## 4 Discussion

To promote HPM in small companies, we developed an outreach-based support strategy through the PPP and assessed its impact, focusing on several implementation outcomes in real-world settings. The Linkworkers achieved successful outreach to small-scale companies; the rate of HPM certification has significantly increased in small companies compared with large companies at both the intervention and community level. Companies that obtained HPM certification implemented WHPPs 2–4 times more frequently when compared with non-certified companies, with a particularly pronounced effect observed in small companies. The retention rate of HPM certification for small companies after 24 months was comparable to that for large companies. Additionally, the Linkworkers themselves, being employees of private enterprises, evaluated this strategy as having a positive impact on their own businesses. Therefore, the Y-Link Project’s outreach strategy successfully enhanced the HPM in small enterprises in Yokohama City, enabling long-term health promotion programs to be implemented. In addition, this strategy aligned with business interests, garnering company support for the interventions.

To our knowledge, this is the first study to demonstrate that the social implementation of the PPP framework can promote HPM and improve the long-term implementation of WHPPs in small companies. Of particular note is that the intervention effectiveness of Linkworkers was more pronounced in small enterprises than in large ones. If implemented on a broader scale and for an extended duration, this strategy could contribute to the reduction of health inequalities based on company size and have a significant impact on public health.

Prior studies have explored practical interventions meant to promote occupational health services in small and medium-sized enterprises (SMEs), albeit to a limited extent. For instance, Al-Khudairy L et al. revealed through a cluster-randomized controlled trial that providing organizational-level financial incentives was associated with increased engagement in health and well-being activities among SMEs (24). However, while employer awareness and behaviors changed, employee behaviors did not shift as expected, suggesting the necessity of organizational support alongside financial incentives. Moreover, financial incentives require substantial funding, raising concerns about the sustainability of such interventions.

Ahonen G et al. reported that positioning multiple SMEs as virtual joint companies could provide access to occupational health services akin to those available to large enterprises (25). However, access to occupational health services for Japanese SMEs is facilitated through the Japan Health Insurance Association to which most SMEs are already enrolled. Nevertheless, disparities in health access based on company size persist.

Saito J et al. emphasized, through meticulous interview research, the pivotal role of employer leadership and motivation in promoting health promotion activities in SMEs (26). Hence, beyond preparing occupational health services, there arises a necessity for establishing a social framework to facilitate “links” between these services and SMEs, particularly with employers.

In our study, interventions by Linkworkers had a more pronounced impact on small-scale enterprises compared to medium-sized or large enterprises. Their effectiveness in advancing health promotion in small-scale companies may be explained by their existing relationships with small business owners, which they cultivated through regular engagement in insurance sales and counseling.

To ensure the successful implementation and sustainability of the new framework, it is essential that stakeholders can reap benefits from it. Given that this study is based on a PPP, it is imperative that the framework offers benefits to the public and private sectors. In this PPP framework, the potential for contributing to cost reduction in healthcare expenditures for the public sector and insurance payout costs for insurance companies was identified through improvements in the health status of community residents and clients. Thus, this strategy managed to avoid the often-problematic issue of conflicting interests in PPPs, allowing for the sharing of benefits derived from the framework. Additionally, Linkworkers themselves recognized the benefits of their activities for their work. This realization likely contributed to their proactive engagement, evidenced by an average reach of seven companies per Linkworker within 2 months, intervention implementation in 4.5 companies, and successful certification acquisition in 3.2 cases. Recently, the “Creating Shared Value” management concept, aiming to achieve economic and social values, has been attracting the attention of health promotion sectors (27). It is speculated that the mutual understanding of this management concept by public and private entities in the future could lead to the development of sustainable PPP frameworks.

Nonetheless, this study revealed some challenges associated with the devised strategy. First, the sustainability of the effectiveness of Linkworkers’ support was identified as a concern. At 6 months after the intervention, certified companies showed a fourfold higher workplace health promotion program implementation rate compared with non-certified companies, but this increase had declined after 18 months. The lack of systematic enforcement of post-intervention follow-up support for intervention companies raised concerns regarding the longevity of the project’s positive impacts. Relying solely on the conduct of training sessions among Linkworkers was insufficient for sustaining long-term effectiveness, emphasizing the need for management policies at the organizational level.

Second, the perceived burden at the operational level emerged as another challenge. Linkworkers cited “burden on primary operations,” “lack of time,” and “insufficient understanding of customer needs” as key issues. It is therefore necessary to implement improvement measures to alleviate the burden at the operational level, such as streamlining the electronic application process and developing user-friendly tools for clients to enhance their comprehension.

This study has several limitations. First, we were unable to conduct a pre-intervention survey because the intervention companies could not be set up in advance. Consequently, the possibility that certified companies were already actively implementing health promotion before the intervention cannot be ruled out. However, considering the lack of difference in the level of awareness of HPM, any disparities in health promotion status prior to the intervention may have been minimal. Second, both follow-up surveys had low response rates. Although no significant differences were found in the characteristics between responding and non-responding companies, the non-responding group included a considerable number of non-certified companies (Supplementary Tables S1, S2). Therefore, the non-responding companies were less receptive to worksite health promotion, potentially leading to an underestimation of the apparent difference in WHPP implementation rates between certified and non-certified companies. In other words, the intervention’s impact may have been underestimated. Finally, because we focused on the characteristics of the companies in this study, we did not address factors related to the employees. However, employee characteristics are important confounding factors that ideally should be adjusted for. Additionally, the impact on employee health status is an important outcome. Therefore, future research should evaluate the effects of this intervention on individual health status, adjusting for factors related to employees such as employee demographics, job types, and working conditions.

In conclusion, our findings showed that the Y-Link Project’s outreach strategy successfully enhanced health and productivity management in small enterprises in Yokohama City, enabling long-term health promotion programs to be implemented. In addition, this strategy aligned with business interests, garnering company support for the interventions, addressing disparities in program availability related to company size.

## Data availability statement

The datasets presented in this article are not readily available because the dataset for the Y-Link Project is not available due to obligations related to the protection of participant data. Requests to access the datasets should be directed to y-kai@my-zaidan.or.jp.

## Ethics statement

The studies involving humans were approved by the ethics committee of the Physical Fitness Research Institute, Meiji Yasuda Life Foundation of Health and Welfare. The studies were conducted in accordance with the local legislation and institutional requirements. Written informed consent to participate in this study was not required from the participants in accordance with the national legislation and the institutional requirements.

## Author contributions

YK: Conceptualization, Data curation, Funding acquisition, Investigation, Methodology, Project administration, Supervision, Visualization, Writing – original draft, Writing – review & editing. YF: Writing – review & editing, Data curation, Investigation, Methodology. NT: Data curation, Formal analysis, Investigation, Methodology, Writing – review & editing. KY: Conceptualization, Investigation, Methodology, Project administration, Visualization, Writing – review & editing. YM-N: Data curation, Formal analysis, Investigation, Methodology, Project administration, Visualization, Writing – review & editing. TN: Conceptualization, Data curation, Investigation, Project administration, Supervision, Writing – review & editing. TJ: Conceptualization, Data curation, Investigation, Methodology, Project administration, Writing – review & editing. TK: Conceptualization, Data curation, Formal analysis, Investigation, Methodology, Writing – review & editing. YY: Data curation, Methodology, Project administration, Supervision, Writing – review & editing. JK: Conceptualization, Data curation, Investigation, Project administration, Supervision, Writing – review & editing. TA: Conceptualization, Funding acquisition, Supervision, Writing – review & editing.

## References

[1] Rongen A Robroek SJW van Lenthe FJ Burdorf A. Workplace health promotion: a meta-analysis of effectiveness. Am J Prev Med. (2013) 44:406–15. doi: 10.1016/j.amepre.2012.12.007 23498108

[2] Hannon PA Garson G Harris JR Hammerback K Sopher CJ Clegg-Thorp C. Workplace health promotion implementation, readiness, and capacity among midsize employers in low-wage industries: a national survey. J Occup Environ Med. (2012) 54:1337–43. doi: 10.1097/JOM.0b013e3182717cf2, PMID: 23090160 PMC3493879

[3] Linnan LA Cluff L Lang JE Penne M Leff MS. Results of the workplace health in America survey. Am J Health Promot. (2019) 33:652–65. doi: 10.1177/0890117119842047 31007038 PMC6643274

[4] McCoy K Stinson K Scott K Tenney L Newman LS. Health promotion in small business: a systematic review of factors influencing adoption and effectiveness of worksite wellness programs. J Occup Environ Med. (2014) 56:579–87. doi: 10.1097/JOM.0000000000000171, PMID: 24905421 PMC4471849

[5] Weinstein M Cheddie K. Adoption and implementation barriers for worksite health programs in the United States. Int J Environ Res Public Health. (2021) 18:12030. doi: 10.3390/ijerph182212030, PMID: 34831782 PMC8622247

[6] Hoshuyama T Hino Y Kayashima K Morita T Goto H Minami M. Inequality in the health status of workers in small-scale enterprises. Occup Med (Lond). (2007) 57:126–30. doi: 10.1093/occmed/kql157 17229721

[7] Kanamori S Tsuji T Takamiya T Kikuchi H Inoue S Takagi D. Size of company of the longest-held job and mortality in older Japanese adults: a 6-year follow-up study from the Japan Gerontological evaluation study. J Occup Health. (2020) 62:e12115. doi: 10.1002/1348-9585.12115, PMID: 32515877 PMC7176136

[8] Goetzel RZ Fabius R Roemer EC Kent KB Berko J Head MA. The stock performance of American companies investing in a culture of health. Am J Health Promot. (2019) 33:439–47. doi: 10.1177/0890117118824818, PMID: 30700099

[9] Fabius R Loeppke RR Hohn T Fabius D Eisenberg B Konicki DL. Tracking the market performance of companies that integrate a culture of health and safety: an assessment of corporate health achievement award applicants. J Occup Environ Med. (2016) 58:3–8. doi: 10.1097/JOM.0000000000000638, PMID: 26716842

[10] Goetzel RZ Ozminkowski RJ Pelletier KR Metz RD Chapman LS. Emerging trends in health and productivity management. Am J Health Promot. (2007) 22:suppl 1-7, iii. doi: 10.4278/0890-1171-22.1.tahp-1 17894264

[11] Mori K Nagata T Nagata M Okahara S Odagami K Takahashi H. Development, success factors, and challenges of government-led health and productivity management initiatives in Japan. J Occup Environ Med. (2021) 63:18–26. doi: 10.1097/JOM.0000000000002002, PMID: 32826547 PMC7773166

[12] American College of Occupational and Environmental Medicine. Excellence in corporate health achievement award (2023). Available at: https://acoem.org/About-ACOEM/Excellence-in-Corporate-Health-Achievement-Award (Accessed April 19, 2023). 10.1097/00043764-199610000-00005 9053135

[13] Corporate Health Standards (2023). Available at: https://nwssp.nhs.wales/a-wp/governance-e-manual/living-public-service-values/corporate-health-standards/ (Accessed April 19, 2023).

[14] United Nations World population prospects 2022: Summary of results (2022). Available at: https://www.un.org/development/desa/pd/content/World-Population-Prospects-2022 (Accessed April 19, 2023).

[15] Ministry of Economy, Trade and Industry. Announcement of organizations selected under the 2022 certified health & productivity management outstanding organizations recognition program (2022). Available at: https://www.meti.go.jp/english/press/2022/0309_002.html (Accessed April 19, 2023).

[16] Takahashi H Nagata M Nagata T Mori K. Association of organizational factors with knowledge of effectiveness indicators and participation in corporate health and productivity management programs. J Occup Health. (2021) 63:e12205. doi: 10.1002/1348-9585.12205, PMID: 33570230 PMC7876858

[17] Wada H Yasuda Y. Value effect of health and productivity management: an event study of the HPM award in Japan. J Occup Environ Med. (2022) 64:465–9. doi: 10.1097/JOM.0000000000002517 35166260

[18] Thornton M Hammerback K Abraham JM Brosseau L Harris JR Linnan LA. Using a social capital framework to explore a broker’s role in small employer wellness program uptake and implementation. Am J Health Promot. (2021) 35:214–25. doi: 10.1177/0890117120957159, PMID: 32914635

[19] Parker LA Zaragoza GA Hernandez-Aguado I. Promoting population health with public-private partnerships: where’s the evidence? BMC Public Health. (2019) 19:1438. doi: 10.1186/s12889-019-7765-2, PMID: 31675935 PMC6824113

[20] Hernandez-Aguado I Zaragoza GA. Support of public-private partnerships in health promotion and conflicts of interest. BMJ Open. (2016) 6:e009342. doi: 10.1136/bmjopen-2015-009342, PMID: 27091816 PMC4838703

[21] Joudyian N Doshmangir L Mahdavi M Tabrizi JS Gordeev VS. Public-private partnerships in primary health care: a scoping review. BMC Health Serv Res. (2021) 21:4. doi: 10.1186/s12913-020-05979-9, PMID: 33397388 PMC7780612

[22] Shelton RC Chambers DA Glasgow RE. An extension of RE-AIM to enhance sustainability: addressing dynamic context and promoting health equity over time. Front Public Health. (2020) 8:134. doi: 10.3389/fpubh.2020.00134, PMID: 32478025 PMC7235159

[23] Ishimaru T Nagata M Hino A Yamashita S Tateishi S Tsuji M. Workplace measures against COVID-19 during the winter third wave in Japan: company size-based differences. J Occup Health. (2021) 63:e12224. doi: 10.1002/1348-9585.12224, PMID: 33955633 PMC8100948

[24] Al-Khudairy L Akram Y Watson SI Kudrna L Hofman J Nightingale M. Evaluation of an organisational-level monetary incentive to promote the health and wellbeing of workers in small and medium-sized enterprises: a mixed-methods cluster randomised controlled trial. PLOS Glob Public Health. (2023) 3:e0001381. doi: 10.1371/journal.pgph.0001381 37410723 PMC10325111

[25] Ahonen G Näsman O Aboagye E. Virtual joint companies as a means of incentivizing SMEs to use occupational health services—a trial in two municipalities in Finland from 2009 to 2011. Front Sustain. (2022) 3:926016. doi: 10.3389/frsus.2022.926016

[26] Saito J Odawara M Takahashi H Fujimori M Yaguchi-Saito A Inoue M. Barriers and facilitative factors in the implementation of workplace health promotion activities in small and medium-sized enterprises: a qualitative study. Implement Sci Commun. (2022) 3:23. doi: 10.1186/s43058-022-00268-4, PMID: 35236511 PMC8889638

[27] Smith D Kramer M Bumb N Silten B. Creating shared value to advance health equity. Am J Health Promot. (2022) 36:1047–50. doi: 10.1177/08901171221092576b, PMID: 35699328

