# Supplementary material for: Promoting health and productivity management in small companies through outreach-based public-private partnership: the Yokohama Linkworker Project
Source: Front Public Health. 2024 Jul 5;12:1345771. doi: 10.3389/fpubh.2024.1345771 (PMC11257930; doi:10.3389/fpubh.2024.1345771)
Supplement: Supplementary file 1 [file Data_Sheet_1.PDF]

Supplementary Table 1. Comparison of characteristics of responded and non-responded companies in 6-month follow-up survey

	Responded companies n=203	Non-responded companies n=158	***P* Value**[1]
Company size			0.22
Micro (<5 employees)	25 (12.3)	34 (21.5)	
Small (5–49 employees)	101 (49.8)	69 (43.7)	
Medium (50–299 employees)	49 (24.1)	34 (21.5)	
Large (≥300 employees)	8 (3.9)	7 (4.4)	
Unknown	20 (9.9)	14 (8.9)	
Industry type			0.55
Manufacturing	28 (13.8)	25 (15.8)	
Service	27 (13.3)	26 (16.5)	
Construction	28 (13.8)	24 (15.2)	
Transportation/postal business	25 (12.3)	11 (7.0)	
Other	79 (38.9)	57 (36.1)	
Unknown	16 (7.9)	15 (9.5)	
Employment type			0.40
Mainly full-time employees	153 (75.4)	120 (75.9)	
Half are non-regular	25 (12.3)	20 (12.7)	
Mainly non-regular employees	3 (1.5)	6 (3.8)	
Unknown	22 (10.8)	12 (7.6)	
Y-HPM Certification[2] in 2021			<0.01
Certified companies	150 (73.9)	69 (43.7)	
Non-certified companies	53 (26.1)	89 (56.3)	

[1] Comparison of certified and non-certified companies using the chi-square test
[2] Y-HPM Certification, Yokohama Health and Productivity Management Certification

Supplementary Table 2. Comparison of characteristics of responded and non-responded companies in 18-month follow-up survey

	**Responded companies** n=203	**Non-responded companies** n=158	***P* Value**[1]
Company size			0.36
Micro (<5 employees)	23 (14.0)	27 (17.0)	
Small (5–49 employees)	89 (54.3)	68 (42.8)	
Medium (50–299 employees)	35 (21.3)	42 (26.4)	
Large (≥300 employees)	6 (3.7)	8 (5.0)	
Unknown	11 (6.7)	14 (8.8)	
Industry type			0.08
Manufacturing	18 (11.0)	32 (20.1)	
Service	21 (12.8)	29 (18.2)	
Construction	25 (15.2)	19 (11.9)	
Transportation/postal business	23 (14.0)	13 (8.2)	
Other	66 (40.2)	54 (34.0)	
Unknown	11 (6.7)	12 (7.5)	
Employment type			0.91
Mainly full-time employees	126 (76.8)	123 (77.4)	
Half are non-regular	21 (12.8)	22 (13.8)	
Mainly non-regular employees	5 (3.0)	3 (1.9)	
Unknown	12 (7.3)	11 (6.9)	
Y-HPM Certification[2] in 2021			0.07
Certified companies	112 (68.3)	92 (57.9)	
Non-certified companies	52 (31.7)	67 (42.1)	

[1] Comparison of certified and non-certified companies using the chi-square test

[2] Y-HPM Certification, Yokohama Health and Productivity Management Certification

This is a provisional file, not the final typeset article
